# Momentary pain assessments reveal benefits of endoscopic discectomy: a prospective cohort study

**DOI:** 10.1097/PR9.0000000000000906

**Published:** 2021-03-17

**Authors:** Kenta Wakaizumi, Binbin Wu, Shishi Huang, Linyu Fan, Bangli Shen, Bo Wu, Jing Zhang, Marwan N. Baliki, A. Vania Apkarian, Lejian Huang

**Affiliations:** a Shirley Ryan AbilityLab, Chicago, IL, USA; b Department of Physical Management and Rehabilitation, Feinberg School of Medicine, Northwestern University, Chicago, IL, USA; c Center for Translational Pain Research, Feinberg School of Medicine, Northwestern University, Chicago, IL, USA; Departments of d Pain Medicine,; e Neurology, and; f Information, the Second Affiliated Hospital and Yuying Children's Hospital of Wenzhou Medical University, Wenzhou, Zhejiang, China; g Department of Physiology, Feinberg School of Medicine, Northwestern University, Chicago, IL, USA

**Keywords:** Lumbar disc herniation, Percutaneous endoscopic lumbar discectomy, Momentary pain rating, Exponential pain reduction model, Pain App

## Abstract

Lumbar disc herniation (LDH) is a common back disorder that evokes back and/or leg pain. Percutaneous endoscopic lumbar discectomy (PELD) is a minimally invasive surgery for patients with LDH. However, there is little evidence of effectiveness of PELD compared with conservative treatments.

**Objective:**

The goal of this study was to quantify the efficacy of PELD compared with conservative treatments.

**Methods:**

Here, we conducted a prospective observational cohort study using momentary pain assessments via a smartphone app during 3 months following surgery. The trajectories of daily ecological momentary pain assessments were fitted with an exponential model containing two parameters: a pain reduction coefficient and the percentage of persistent pain. To control for selection bias between PELD and Conservative groups (N = 167 and 34), we used inverse probability (IP) of treatment weighting for statistical comparisons.

**Results:**

Compared with conservative treatments, both momentary pain rating and the exponential model showed statistically significant pain recovery following PELD (p < 0.001). In addition, PELD had a faster pain recovery rate (hazard ratio (95% confidence interval): 1.75 (1.40, 2.20), p < 0.001), greater overall pain recovery rate (odds ratio (95%CI): 2.35 (2.01, 5.26), p < 0.001), faster pain reduction (t_199_ = 3.32, p = 0.001), and lower estimated persistent pain (Z = 2.53, p = 0.011). Greater pain intensity and lower anxiety before the surgery were predictors of faster pain reduction in the recovery subgroup following PELD.

**Conclusions:**

In conclusion, momentary pain rating and the model fitting revealed that PELD provided rapid pain recovery that lasted for at least three months. Greater pain intensity and lower anxiety before the surgery were predictors of faster pain reduction in the recovery subgroup following PELD. Daily momentary pain rating on a smartphone may be able to provide more informative data to evaluate effect of an intervention than pain assessment on hospital visits.

## Abstract 2

Both pain rating and exponential model revealed that percutaneous endoscopic lumbar discectomy provided rapid pain recovery that was maintained for at least 3 months compared with conservative treatments.

## 1. Introduction

Lumbar disc herniation (LDH) is a common back disorder, in which a portion of the intervertebral disc breaks out to the surrounding space. The herniated tissues compress and irritate nearby nerves via inflammation, resulting in lower back pain and/or typical sciatica, numbness, and weakness of the lower extremities.^2,25^ The overall prevalence of symptomatic LDH is approximately 1% to 3% of the population in the United States and Europe,^24,36^ and 7.62% in a province in China.^22,37^

Lumbar discectomy is the most popular surgery for patients with LDH, which includes removal of the herniation and decompression of the injured tissues to provide pain relief and functional recovery. In recent years, technological developments have considerably improved lumbar discectomies by minimizing the burden on patients and reducing postoperative complications. In particular, percutaneous endoscopic lumbar discectomy (PELD) is one such procedure, which is performed using a single port of an endoscopic surgical system. However, previous methodological approaches have limited the interpretability of studies on PELDs,^16^ and as a result, there is little evidence quantifying the efficacy of PELD compared with conservative treatments.^32^

Indeed, LDH is often observed in imaging studies on asymptomatic patients,^6^ and can spontaneously regress over time without surgery.^12^ In addition, 90% of sciatica cases attributed to LDH subside with conservative management.^23^ These data support the use of conservative treatments for symptomatic patients. Furthermore, several observational cohort studies and randomized control trials, which compared the effectiveness of surgical vs conservative treatments in symptomatic patients,suggest improvements in short-term but not long-term pain recovery after both open^39^ and micro-endoscopic discectomy.^1^ Hence, incorporating the fact that conservative treatments have a lower risk of complications than surgery,^11^ some clinicians cast doubt on the net benefit of surgical treatment.

Clinical observational cohort studies are limited by the presence of systematic baseline differences between groups. Confounding by indication often renders results difficult to interpret. Although such biases can be minimized with randomized control trials, a large proportion of patients randomly allocated to a conservative group may eventually choose to receive surgical treatment,^30,39^ suggesting randomized groups can differ from those in the clinical setting. To evaluate representative clinical outcomes while controlling for confounds, we conducted a large observational cohort study in a standard clinical setting, and controlled for baseline imbalances using propensity scores—inverse probability (IP) of treatment weighting.^3^

An important limitation of clinical pain research is unstable pain ratings, which typically fluctuate on the scale of hours and days. Such volatility is not typically captured, and pain is usually assessed only when participants visit the office. Even if patients are asked to provide a rating of average pain during the last few days or weeks, recall bias will contaminate outcomes.^5^ Thus, in this work, we obtained daily momentary assessments of pain using a smartphone app, which provides more ecologically appropriate (natural setting of daily life) and reliable momentary pain ratings during the observational period.

## 2. Materials and methods

### 2.1. Participants

We recruited 240 low back and/or leg pain patients with lumbar disc herniation (LDH) from June 2017 to April 2019 in Wenzhou, China. To be eligible, subjects must (1) be older than 18 but younger than 75 years of age; (2) have a diagnosis of lumbar disc herniation (LDH) diagnosed by medical history, physical examination, and consistent MRI assessment confirmed independently by 2 radiologists; and (3) have leg and/or back pain that persisted for at least 12 weeks. Participants were excluded if they (1) were younger than 18 or older than 75 years of age; (2) had diabetes and psychiatric disease (may affect pain ratings); (3) reported history of brain neurosurgical procedures and/or epilepsy; (4) were unable to cooperate (eg, psychogenic or cognitively impaired); (5) reported drug dependence or abuse; and (6) underwent any surgery for LDH other than PELD.

This study was approved by the Institutional Review Board of the Second Affiliated Hospital and Yuying Children's Hospital of Wenzhou Medical University, China (Approval number: Clinical Scientific Research Ethical Review No. 8-2017), and all participants signed a written informed consent. All procedures were performed in accordance with the Declaration of Helsinki, International Conference on Harmonization-Good Clinical Practice, the China Food and Drug Administration-Good Clinical Practice guidelines, and relevant laws and regulations in China.

### 2.2. Study design

**Figure 1.**
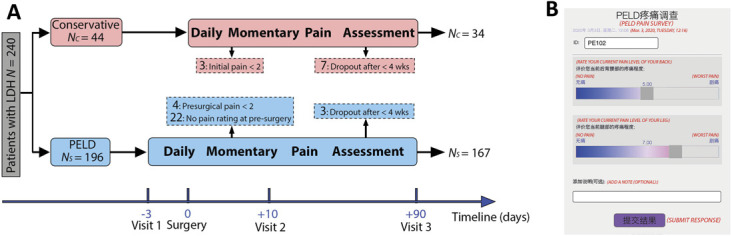
Chart of study flow and electronic smartphone application for collecting pain intensity. (A) Two hundred forty patients with LDH participated in this study, of whom 196 underwent percutaneous endoscopic lumbar discectomy (PELD) (PELD group) and 44 were treated with conservative therapies (Conservative group). One hundred sixty-seven and 34 participants in both groups remained for analysis. Each participant started to report his/her pain intensity twice per day via a smartphone app following baseline screening (timeline: −3, visit 1); this daily momentary pain assessment continued until the last onsite follow-up (timeline: +90, visit 3). (B) The interface of the smartphone app. The red italicized words in parenthesis represent the translation of original Chinese and were not displayed on the app. Participants entered their assigned ID and then rated their pain level of their back and leg on a scale from 0 to 10. The app also had an optional note for participants to fill comments or questions.

Two hundred forty LDH participates were recruited, of whom 196 underwent PELD surgery (PELD group) and 44 were treated with conservative therapies (Conservative group) (Fig. 1A). Each participant in the PELD group started to report his/her pain intensity twice per day via a smartphone app (see 2.5. Electronic Smartphone App) after baseline screening (timeline: −3, visit 1) and this daily momentary pain assessment continued until the last onsite follow-up (timeline: +90, visit 3). Twenty-nine participants in the PELD group were excluded (4 participants who reported presurgical pain intensity less than 2, 22 participants without pain ratings before surgery, and 3 participants who dropped out within 4 weeks). So, 167 participants in the PELD group remained for data analysis, of whom all 167 participants completed self-report questionnaires at baseline (timeline: −3, visit 1), and 73 completed the same questionnaires in 2 follow-up visits (timeline: +10, visit 2 and +90, visit 3), respectively (see 2.3 Behavioral measures).

For the Conservative group, each participant reported his/her pain intensity twice per day via the app directly after baseline screening. For the purpose of simplicity, the starting date was adjusted and aligned to *t* = 0, Surgery, and the daily momentary pain assessment continued until the last onsite follow-up (timeline: +90, visit 3) as well. Ten participants in the Conservative group were excluded from this study (3 participants reported initial pain intensity less than 2, and 7 participants who dropped out within 4 weeks). In the end, 34 participants remained for analysis, of whom 34 completed self-report questionnaires during baseline screening (timeline: 0, Surgery), and 17 completed the same questionnaires in 2 follow-up visits (timeline: +10, visit 2 and +90, visit 3), respectively.

### 2.3. Behavioral measures

All participants completed a demographic survey asking about their age, sex, education level, dominant pain location (low back or leg), and pain duration at baseline screening (Fig. 1A, timeline: −3, visit 1). Education was categorized to low education (middle school or below) and high education (high school or above).

In addition, a battery of self-report questionnaires related to pain were completed during baseline screening (Fig. 1A, Timeline: 0, Surgery) and at 2 follow-up visits (Fig. 1A, timeline: +10, visit 2 and +90, visit 3), items included Numerical Rating Scale (NRS),^14^ Oswestry Disability Index (ODI),^13,26^ McGill Pain Questionnaire (MPQ)—Short Form,^8,20^ Pain Catastrophizing Scale (PCS),^34,35^ Pain Anxiety Symptoms Scale (PASS),^28,42^ Positive and Negative Affect Schedule (PANAS),^21,38^ Beck Depression Inventory (BDI),^4,41^ and Pain Sensitivity Questionnaire (PSQ).^31,33^ Numerical Rating Scale is an 11-point NRS used to measure pain intensity, where 0 corresponds to no pain and 10 indicates worst pain possible (or imaginable). Oswestry Disability Index assesses physical impairment in relation to pain; MPQ—Short Form is a well-validated pain measure with affective and sensory components of pain (MPQ/a and MPQ/s), which also includes a visual analogue pain scale of pain. Pain Catastrophizing Scale is a 5-point instrument to assess 13 thoughts or feelings related to past pain experiences. Pain Catastrophizing Scale yields 3 subscale scores assessing rumination (PCS/r), magnification (PCS/m), and helplessness (PCS/h). Pain Anxiety Symptoms Scale measures fear and anxiety responses specific to pain. The PASS consists of 4 aspects of pain-related anxiety: cognitive suffering (PASS/c), escape-avoidance behaviors (PASS/e), fear of pain (PASS/f), and physiological symptoms of anxiety (PASS/p). Positive and Negative Affect Schedule has 2 mood scales, one measuring positive affect (PANAS/p) and the other measuring negative affect (PANAS/n). Each scale is rated on a 5-point, 10-item scale. Beck Depression Inventory is a 21-item instrument for measuring the severity of depression. Pain Sensitivity Questionnaire is an 11-point, 17-item instrument used to assess individual pain sensitivity. Pain Sensitivity Questionnaire is based on pain intensity ratings of hypothetical situations, which include various modalities (heat, cold, pressure, and pinprick) and measures (pain threshold and intensity ratings). Pain Sensitivity Questionnaire-minor (PSQ/min) and PSQ-moderate (PSQ/mod) were 2 subscales corresponding to mildly painful and moderately painful situations, respectively.

All questionnaires were administered in Chinese and collected on an electronic tablet device using Research Electronic Data Capture.^18^ Research Electronic Data Capture is a secure, convenient, and efficient web application for capturing electronic survey data.

### 2.4. Treatment interventions

The assignment to treatment interventions (PELD or Conservative) was determined by patients and their physicians together after the patients were fully informed of their clinical indications. Following the treatment recommendations of LDH in Ref. 25, the physicians would suggest a treatment intervention. Patients who were economically disadvantaged and whose activities of daily living were not severely affected by pain were more likely to choose conservative treatments.

All PELD surgeries were conducted by 3 well-trained surgeons in Department of Pain Medicine of the Second Affiliated Hospital and Yuying Children's Hospital of Wenzhou Medical University, China, following the standard procedure of Transforaminal Endoscopic Spine System (Joimax GmbH, Karlsruhe, Germany). Briefly, the surgery consisted of the following steps in sequence: (1) determine affected discs and pedicles under fluoroscopic guidance; (2) perform subcutaneous local anesthesia and insert a needle to reach the distal segmental superior articular process; (3) insert a guide wire along the needle and make a circle incision followed by serial dilations and forward rotation of working channel along the guide wire; (4) insert a guide bar after removing the guide wire; and (5) remove part of the cartilage of superior articular process and perform endoscopic foraminoplasty to allow the working channel to access to herniated disc for complete decompression. The participants who complained of pain after PELD took nonsteroidal anti-inflammatory drugs for pain relief.

The conservative treatment interventions were determined on a case-by-case basis, including physical therapy, Chinese herbal medicine, activity modification, epidural steroid injection, and nonsteroidal anti-inflammatory drugs for pain relief.

### 2.5. Electronic Smartphone App

Using an app (Fig. 1B) installed in participants' smartphones at baseline screening, participants rated their pain intensity of their back and legs twice per day (morning and afternoon/evening) for the duration of the study. Pain ratings were on a scale from 0 to 10, with 0 being no pain and 10 being the worst pain imaginable. The app also had an optional note section. All ratings were sent to a secure server and stamped with the date and time completed. A check-missing script was run on the server daily and would send a warning message to a project coordinator if a participant missed 2 ratings within 2 days, who would contact and remind the participant to complete their ratings. With the assistance of this check-missing script and the efforts made by the project coordinator, the missing rate of daily pain ratings was less than 5% on the whole.

### 2.6. Exponential model for fitting pain trajectory

The 2 daily ratings were averaged to render a daily momentary pain rating for that day. For analyses, pain intensity of each time point was expressed as a percentage of pre-surgery (PELD group) or initial (Conservative group) pain intensity at day 0 (timeline: 0, Surgery in Fig. 1A). A 2-parameter exponential pain reduction model was introduced to fit the trajectory of daily momentary pain rating as follows:

(1) $$\text{Pain\%}\left(\text{t}|{\text{P}}_{\text{p}},\text{λ}\right)=\left(100-{\text{P}}_{\text{p}}\right)\times \text{exp}\left(-\text{λ}\times \text{t}\right)+{\text{P}}_{\text{p}}$$

where Pain%, t, P_p_, and λ represent percentage of initial pain intensity, time in units of day, persistent pain in units of percentage, and pain reduction coefficient in units of 1/day, respectively; t, P_p_, and λ are nonnegative; 1/λ is the time constant of the exponential model. The 2 parameters in the Equation 1, P_p_ and λ, were estimated using nonlinear least squares (MATLAB function *lsqcurvefit*).^9^

In our study, we defined *recovery* as 70% reduction of pain intensity relative to initial pain. Therefore, an *overall recovery* was identified if persistent pain, P_P_ in the Equation 2, is less than 30. In addition, recovery time (T_R_), defined as day(s) to achieve at 30% of initial pain intensity, was

(2) $$T_{R}=\left\{ \begin{array}{l} \frac{ln\left(100-P_{p}\right)-ln\left(30-P_{p}\right)}{\lambda }\;P_{p}<30\\ \infty \;P_{p}\geq 30 \end{array}\right.$$

### 2.7. Statistical analysis

The data analysis and statistical plan was written after the data were accessed.

To account for selection bias due to baseline differences between 2 groups, each individual was weighted using the IP of treatment. The probability (also called propensity score, PS) was estimated from a logistic regression model, given the independent variables from baseline, including age, sex, low education, leg-pain dominant patients, log-transformed pain duration, NRS, MPQ/vas, MPQ/a, MPQ/s, ODI, PCS/r, PCS/m, PCS/h, PASS/f, PASS/p, PASS/c, PASS/e, PANAS/n, PANAS/p, BDI, PSQ/min, and PSQ/mod (Table 1). The weights were 1/PS for the participants in the PELD group and 1/(1−PS) for the participants in the Conservative group. As a consequence, results from IP-weighted analyses can be interpreted similarly to a randomized trial without selection bias.^27^

**Table 1:** Comparison of demographics and pain characteristics between PELD and Conservative groups at baseline.

	Conservative (N = 34)	PELD (N = 167)	*P*	Adjusted *P*
Age (y/o), mean (SD)	41.9 (12.2)	44.0 (11.8)	0.359	0.647
Male, *N* (%)	16 (47.1)	109 (65.2)	**0.049**	0.505
Low education, *N* (%)	17 (50.0)	110 (65.9)	0.085	**0.009**
Leg-pain dominant patient, *N* (%)	16 (47.1)	135 (80.8)	**< 0.001**	0.186
Pain duration (wk), median (min, max)	100 (12, 520)	104 (2, 1040)	0.532*	0.695*

	Mean (SD)	Mean (SD)	*P*-value	Adjusted *P*-value
Pain-related measures				
NRS	4.5 (1.6)	5.0 (1.9)	0.101	0.456
MPQ/vas	43.6 (15.6)	50.2 (18.2)	0.050	0.386
MPQ/a	6.0 (2.2)	6.9 (2.6)	**<0.001**	0.055
MPQ/s	15.9 (5.1)	16.1 (3.8)	0.059	0.967
ODI	14.9 (7.1)	22.1 (9.8)	0.757	0.201
PCS/r	8.7 (3.3)	8.1 (3.2)	0.326	0.720
PCS/m	4.6 (2.9)	4.0 (2.4)	0.233	0.764
PCS/h	6.6 (5.8)	6.3 (5.2)	0.694	0.910
PASS/f	8.7 (7.6)	6.9 (5.5)	0.111	0.382
PASS/p	3.4 (5.4)	2.9 (3.9)	0.526	0.207
PASS/c	8.1 (6.2)	7.6 (5.4)	0.655	0.790
PASS/e	11.4 (6.1)	13.6 (6.0)	0.053	0.302
PANAS/n	18.3 (7.1)	16.3 (5.4)	0.168	0.201
PANAS/p	18.9 (6.1)	19.0 (6.8)	0.065	0.051
BDI	9.4 (7.3)	7.7 (6.3)	0.896	0.553
PSQ/min	4.4 (1.8)	3.6 (1.5)	**0.007**	0.135
PSQ/mod	5.8 (1.9)	5.3 (1.8)	0.098	0.320

A statistically significant difference was observed in sex (*P* = 0.049), pain-dominant location (*P* < 0.001), MPQ/a (*P* < 0.001), and PSQ/min (*P* = 0.007). *P*-values were generated using unpaired *t*-tests. Adjusted *P*-values were generated using IP-weighted unpaired *t*-tests. Bold entries represent statistical significance.

* Log-transformation of pain duration was used for the test.

BDI, Beck Depression Inventory; NRS, Numerical Rating Scale; MPQ, McGill Pain Questionnaire; ODI, Oswestry Disability Index; PCS, Pain Catastrophizing Scale; PASS, Pain Anxiety Symptoms Scale; PANAS, Positive and Negative Affect Schedule; PSQ, Pain Sensitivity Questionnaire; PELD, percutaneous endoscopic lumbar discectomy.

Unpaired *t*-tests (for continuous variables) and logistic regressions (for binary variables) were performed to compare participant demographics and pain characteristics between the PELD and Conservative groups at baseline. Inverse probability-weighted unpaired *t*-tests (for continuous variables) and IP-weighted logistic regressions (for binary variables) were performed to detect if IP of treatment weighting properly corrected for selection bias between the PELD and Conservative groups.

To generate a mean normalized pain trajectory for each group, the normalized daily momentary pain ratings across the group were averaged at each day for 90 days, and the group's 95% confidence intervals were calculated accordingly. In the case(s) of missing daily rating(s), the mean from nearest neighboring point was used to replace the missing one(s). In addition, estimated pain was generated from the pain reduction model.

Multiple statistical models were created, and the resulting parameter estimates were compared between the PELD and Conservative groups. First, IP-weighted Cox hazard model was used to compare pain recovery rate, which corresponds to survival rate in a Cox hazard model and is equal to (1—survival rate), between PELD and Conservative groups; IP-weighted logistic regression model was used to compare overall recovery rate, which was defined as the proportion of participants whose estimated persistent pain (P_P_) was 30 or less, between PELD and Conservative groups. After this, an IP-weighted unpaired *t* test was performed to compare log-transformed λ, pain reduction coefficient. In addition, Wilcoxon log-rank test was performed to compare P_P_, estimated persistent pain.

Within- and between-group comparisons of 3 different pain measures at 2 visits (visit 2 and visit 3) were performed (Table 2). There were 3 pain measures in this study: (1) NRS from one of the self-report questionnaires filled at each visit; (2) averaged 3-day pain rating, which was the mean normalized pain rating of the 3 days before each visit; and (3) estimated pain at the visiting day derived from the pain reduction model (Equation 1). Fifty-four subjects in the PELD group and 12 in the Conservative group had all 3 of pain measures. For each visit, a two-tailed paired *t* test with Bonferroni correction for multiple comparisons was used to compare within-group differences between the estimated pain and averaged 3-day pain rating, and between NRS and averaged 3-day pain rating, respectively. With each pain measure, an IP-weighted unpaired *t* test with Bonferroni correction for multiple comparisons was performed to examine the group differences between the PELD and Conservative groups.

**Table 2:** Within- and between-group comparisons of 3 different pain measures at 2 visits.

	Conservative (*N* = 12)				PELD (*N* = 54)				Group difference	
	Mean	SD	*t*-value	*P*-value	Mean	SD	*t*-value	*P*-value	*t*-value	*P*-value
Visit 2 (timeline: +10 d)										
Averaged 3-d pain rating	2.23	1.74	—	—	1.61	1.47	—	—	**−2.54**	0.014*
Estimated pain	2.49	1.99	1.34	0.206	1.35	1.07	−1.80	0.078	**−4.26**	< 0.001*
NRS	2.91	2.14	1.37	0.199	2.02	1.48	**2.51**	0.015*	**−2.82**	0.006*
Visit 3 (timeline: +90 d)										
Averaged 3-d pain rating	1.86	1.74	—	—	1.12	1.25	—	—	−1.71	0.092
Estimated pain	2.17	2.09	0.86	0.401	1.05	1.07	−0.60	0.550	**−4.19**	< 0.001*
NRS	2.89	1.76	2.44	0.033	2.25	1.99	4.33	< 0.001*	−1.08	0.284

Within-group compared differences between the estimated pain and averaged 3-day pain rating, and between NRS and averaged 3-day pain rating, respectively. Between-group compared group difference of 3 pain measures between PELD and Conservative. There was no statistically significant difference between estimated pain from the model and average 3-day pain rating for both PELD and Conservative groups at each visit, but statistically significant differences were observed between NRS and averaged 3-day pain rating for PELD group (*t*_53_ = 2.51, *P* = 0.015 at visit 2 and *t*_53_ = 4.33, *P* < 0.001 at visit 3) and a similar trend for Conservative group (*t*_11_ = 1.37, *P* = 0.199 at visit 2 and *t*_11_ = 2.44, *P* = 0.033 at visit 3). For between-group comparisons, only estimated pain reached statistical significance at both visits (*t*_1,64_ = −4.26, *P* < 0.001 at visit 2 and *t*_1,64_ = −4.19, *P* < 0.001 at visit 3).

—, data unavailable. Bold entries represent statistical significance.

* Bonferroni's corrected *P*-value < 0.05).

NRS, Numerical Rating Scale; PELD, percutaneous endoscopic lumbar discectomy.

In addition, we analyzed demographic characteristics and pain-related measures at baseline to predict the pain reduction coefficient, λ. First, demographics at baseline between the nonrecovery (N = 39) and recovery (N = 128) subgroups in the PELD group were compared using unpaired *t*-tests. Second, pain characteristics at baseline between these subgroups were compared using analysis of covariance with age, sex, and low education at baseline as covariates. Finally, step-wise multivariable regression analysis of log-transformed λ (*P*-value of 0.05 as inclusion and exclusion thresholds, forward and backward direction) with adjustment of age, sex, low education, dominant location of pain, and Log(Pain duration) as covariables was performed to select the baseline pain-related measures associated with λ in the recovery subgroup.

Finally, we investigated group differences of measures related to pain other than NRS. An IP-weighted mixed-effects model was applied to analyze the longitudinal measures from visit 1 to 3, as assessed by the questionnaires including MPQ, ODI, PCS, PASS, PANAS, BDI, and PSQ. The significance of the group-by-time interaction represents the surgical effect of interest.

All statistical analyses were performed using MATLAB 2016a and JMP Pro version 13.2 (SAS Institute, Cary, NC).

## 3. Results

### 3.1. Sensitivity power analysis

We conducted a sensitivity power analysis using G*Power 3.1.^15^ We included 167 participants in the PELD group and 34 in the Conservative group, so we performed the power analysis with this sample size based on the assumption of a 2-tailed comparison and type I error rate set as 0.05 with 80% power. An effect size of 0.53 would be required to meet these constraints, which is close to a moderate effect size (0.5).^7^ Moreover, Bartlett homogeneity test showed that the variances for the original pain rating of the 2 groups are not statistically significantly different (*P* = 0.27).

### 3.2. Comparisons of demographics and pain characteristics at baseline between percutaneous endoscopic lumbar discectomy and conservative groups

As shown in Table 1, unpaired *t*-tests determined that there were important group differences in sex (*P* = 0.049), pain-dominant location (*P* < 0.001), MPQ/a (*P* < 0.001), and PSQ/min (*P* = 0.007) between Conservative and PELD groups measured at baseline (Fig. 1A, timeline: −3, visit 1). For IP-weighted unpaired *t*-tests, only education level was observed to have a statistically significant difference (*P* = 0.009).

### 3.3. Performance of exponential pain reduction model

**Figure 2.**
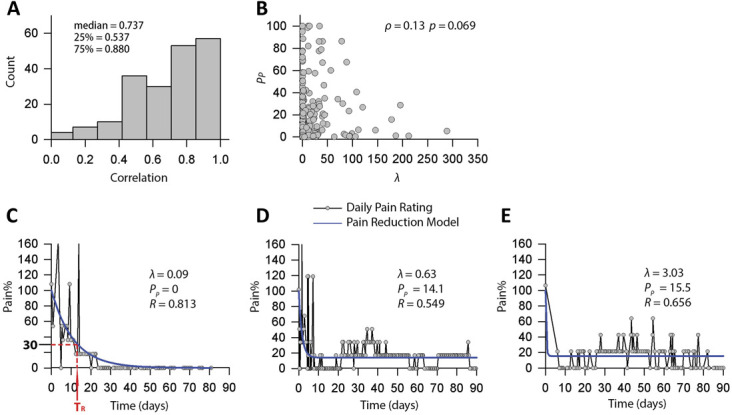
Performance of exponential pain reduction model and 3 cases of normalized pain trajectory and estimated pain by the model. (A) Histogram of correlations (*R*) between the normalized pain trajectory of daily momentary pain ratings and estimated pain. (B) Spearman correlation determined that there was no significant association between 2 estimated parameters in the model, λ, the pain reduction coefficient, and P_P_, persistent pain (Spearman's ρ = 0.13, *P* = 0.069). (C) A representative case of longer Time of Pain Recovery (T_R_) with 0% persistent pain (*λ* = 0.09, *P*_*P*_ = 0, *R* = 0.813). The red arrow with 2 red dotted lines depicted the Recovery Time (T_R_) in Equation 2. (D) A representative case of shorter T_R_ with 14.1% persistent pain (λ = 0.63, P_P_ = 14.1, *R* = 0.549). (E) A representative case of much shorter T_R_ with 15.5% persistent pain (λ = 3.03, P_P_ = 15.5, *R* = 0.656).

The exponential model performed very well; the distribution of correlations between pain estimated by the exponential model and normalized pain intensity rated (ie, predicted vs observed) on the app are shown in Figure 2A. The 25th percentile, median, and 75th percentile of the correlations were 0.537, 0.737, and 0.880, respectively. In addition, Spearman correlations determined that there was no significant association between 2 estimated parameters in the model, λ, the pain reduction coefficient, and P_P_, persistent pain (Spearman's ρ = 0.13, *P* = 0.069) (Fig. 2B**)**. Figure 2C–E illustrates 3 representative cases, from longer (C) to shorter (E) pain recovery time (T_R_). It was observed that with increased λ, the slope of the fitted curves became steeper and the T_R_ became shorter.

### 3.4. Percutaneous endoscopic lumbar discectomy showed statistically significantly greater pain recovery compared with conservative treatments

**Figure 3.**
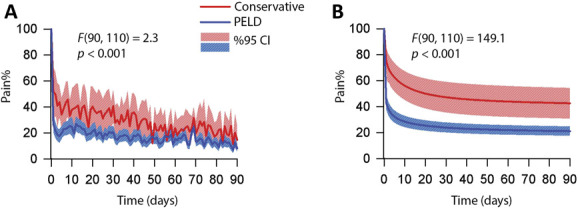
Both momentary pain rating and the exponential pain reduction model showed statistically significant pain recovery after PELD compared with conservative treatments. (A) Group averages of normalized pain trajectory of daily momentary pain rating (solid lines) and its 95% confidence interval (forward diagonal shaded areas) for the PELD group (blue) and Conservative group (red). (B) Group averages of estimated pain generated by the pain reduction model (solid lines) and its 95% confidence interval (forward diagonal shaded areas) for the PELD group (blue) and Conservative group (red). PELD, percutaneous endoscopic lumbar discectomy.

Both momentary pain ratings and the exponential model showed statistically significant pain recovery after PELD compared with conservative treatments. Group averages of normalized pain trajectories of daily momentary pain ratings showed a decreasing trend throughout the observational period after a rapid initial mitigation over the first few days (Fig. 3A). Inverse probability-weighted repeated-measures ANOVA determined that the PELD group had greater pain reduction within 90 days after the surgery when compared with Conservative group (*F*(90,110) = 2.3, *P* < 0.001), whereas the averaged pain of the 2 groups slowly converged as the observation period ended (day 90). However, as shown in Figure 3B, the model smoothed the daily fluctuations and resulted in a greater group difference (*F*(90,110) = 149.1). Furthermore, with the assistance of the model, the PELD effect could be observed in not only the beginning but also at the end of the study (Fig. 3B).

### 3.5. The exponential model indicated rapid and sustained benefits of pain recovery of percutaneous endoscopic lumbar discectomy

**Figure 4.**
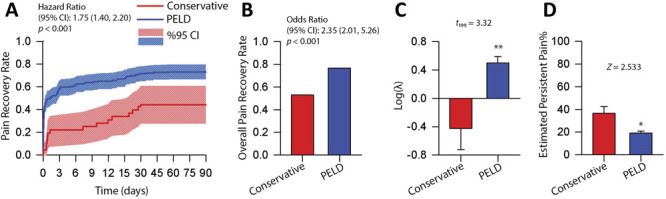
Estimated parameters derived from the exponential model indicated that PELD group was significantly better in reducing pain than conservative treatments. (A) Kaplan-Meier curves of pain recovery rates of the PELD group (blue solid line) and Conservative group (red solid line) and their 95% confidence intervals (forward diagonal shaded areas). (B) Inverse probability-weighted logistic regression analysis of overall pain recovery rate; odds ratio (95% CI) of the PELD was 2.35 (2.01, 5.26), *P* < 0.001; the overall pain recovery rate of PELD and Conservative was 0.767 and 0.529, respectively. (C) λ, which represents rapidness of pain reduction, showed PELD reduced pain faster than Conservative (*t*_199_ = 3.32, *P* = 0.001. (D) PELD had lower estimated persistent pain (P_P_) than Conservative group (*Z* = 2.53, *P* = 0.011. CI, confidence interval; PELD, percutaneous endoscopic lumbar discectomy.

Estimated parameters derived from the exponential model indicated that the PELD group had superior pain reduction when compared to conservative treatments in LDH. Kaplan-Meier curves of pain recovery rates shown in Figure 4A indicated that PELD had greater pain recovery rates than Conservative (hazard ratio [95% confidence interval]: 1.75 (1.40-2.20), *P* < 0.001). In addition, the PELD group showed a greater overall pain recovery rate (odds ratio [95% confidence interval]: 2.35 [2.01-5.26], *P* < 0.001) (Fig. 4B); faster pain reduction, as indicated by log-transformed λ (*t*_199_ = 3.32, *P* = 0.001) (Fig. 4C); and lower estimated persistent pain, P_P_ (*Z* = 2.53, *P* = 0.011) (Fig. 4D). Figure 4C indicates that the time constant for pain relief was on average 0.32 days in the PELD group and 2.64 days in the Conservative group.

### 3.6. Within- and between-group comparisons of 3 different pain measures at 2 follow-up visits

Table 2 shows differences among 3 pain measures within and between both Conservative and PELD groups at 2 visits. There was no statistically significant difference between estimated pain from the model and average 3-day pain rating for both PELD and Conservative groups at each visit, but statistically significant differences were observed between NRS and averaged 3-day pain rating for PELD group (*t*_53_ = 2.51, *P* = 0.015 at visit 2 and *t*_53_ = 4.33, *P* < 0.001 at visit 3) and a similar trend for Conservative group (*t*_11_ = 1.37, *P* = 0.199 at visit 2 and *t*_11_ = 2.44, *P* = 0.033 at visit 3). For between-group comparisons, only estimated pain reached statistical significance at both visits (*t*_1,64_ = −4.26, *P* < 0.001 at visit 2 and *t*_1,64_ = −4.19, *P* < 0.001 at visit 3).

### 3.7. Higher pain intensity and lower anxiety predict rapid response to the percutaneous endoscopic lumbar discectomy

As shown in Table 3, a statistically significant difference was observed only in NRS (*t*_1,165_ = 2.07, *P* = 0.003) and MPQ/vas (*t*_1,165_ = 2.07, *P* = 0.008) between nonrecovery and recovery subgroups in the PELD group at baseline. Moreover, as shown in Table 4, 2 pain-related measures, NRS (*t*_7,120_ = 3.08, *P* = 0.003) and psychological anxiety of PASS, PASS/p (*t*_7,120_ = 3.08, *P* = 0.003), were identified by a step-wise multivariable regression analysis with adjustments of age, sex, low education, dominant location of pain, and Log (Pain duration) as covariates to predict log-transformed λ, implicating higher pain intensity and lower anxiety measured at baseline corresponding to a rapid response to the PELD.

**Table 3:** Comparison of demographics and pain characteristics between nonrecovery (N = 39) and recovery (N = 128) subgroups in PELD groups at baseline (timeline: −3, visit 1).

	Nonrecovery (N = 39)	Recovery (N = 128)	*p*-value
Age (y/o), mean (SD)	42.8 (1.9)	44.4 (1.0)	0.480
Male, N (%)	25 (64.1)	84 (65.6)	0.862
Low education, N (%)	29 (74.4)	81 (63.3)	0.194
Leg-pain dominant patient, N (%)	29 (74.3)	106 (82.8)	0.252
Pain duration (weeks), median (min, max)	100 (12, 520)	104 (2, 1040)	0.613*

	Mean (SD)	Mean (SD)	*p*-value
Pain-related measures			
NRS	4.2 (1.4)	5.3 (2.0)	0.002
MPQ/vas	43.1 (14.8)	52.3 (18.7)	0.005
MPQ/affective pain	7.1 (2.6)	6.8 (2.7)	0.514
MPQ/sensory pain	16.7 (4.5)	15.9 (3.5)	0.253
ODI	19.9 (9.4)	22.7 (9.9)	0.118
PCS/rumination	8.1 (3.2)	8.1 (3.2)	0.967
PCS/magnification	4.1 (2.4)	4.0 (2.3)	0.902
PCS/helplessness	6.4 (4.8)	6.2 (5.4)	0.802
PASS/fear of pain	7.3 (5.7)	6.8 (5.5)	0.571
PASS/physiological symptoms of anxiety	2.8 (3.4)	2.9 (4.0)	0.873
PASS/cognitive suffering	7.9 (5.9)	7.5 (5.3)	0.665
PASS/escape-avoidance behaviors	13.3 (5.5)	13.7 (6.2)	0.735
PANAS/negative	16.7 (5.1)	16.2 (5.4)	0.638
PANAS/positive	20.7 (6.9)	18.5 (6.7)	0.078
BDI	7.1 (5.5)	7.8 (6.6)	0.543
PSQ/minor	3.6 (1.2)	3.6 (1.5)	0.779
PSQ/moderate	5.5 (2.0)	5.2 (1.7)	0.244

A statistically significant difference was observed only in NRS (*t*_1,165_ = 2.07, *P* = 0.003) and MPQ/vas (*t*_1,165_ = 2.07, *P* = 0.008). Two-tailed unpaired *t* test or χ^2^ test was performed for group comparisons of demographics. ANCOVA was performed to compare pain-related measures with age, sex, and low education as covariates.

* Log-transformation of pain duration was used for *t* test.

BDI, Beck Depression Inventory; NRS, Numerical Rating Scale; MPQ, McGill Pain Questionnaire; ODI, Oswestry Disability Index; PCS, Pain Catastrophizing Scale; PASS, Pain Anxiety Symptoms Scale; PANAS, Positive and Negative Affect Schedule; PSQ, Pain Sensitivity Questionnaire; PELD, percutaneous endoscopic lumbar discectomy.

**Table 4:** Baseline measures predict λ in patients with recovery after surgery (N = 128).

Variables	β	SEM	*t*-value	*P*	Model fitting
NRS	0.04	0.01	3.08	**0.003**	*F*(7,120) = 2.51, *P* = 0.019
PASS/p	−0.13	0.06	−2.12	**0.036**	R^2^ = 0.165, RMSE = 2.687
Covariates					
Age	−0.01	0.02	−0.44	0.663	
Sex (male)	0.31	0.25	1.23	0.222	
Low education	0.30	0.28	1.05	0.295	
Dominant location of pain (leg)	−0.43	0.32	−1.35	0.179	
Log(Pain duration)	−0.23	0.41	−0.56	0.579	

Two pain-related measures at baseline, NRS (*t*_7,120_ = 3.08, *P* = 0.003) and PASS/p (*t*_7,120_ = 3.08, *P* = 0.003), were identified as predictors of log-transformed *λ* (β: regression coefficient, SEM: standard error of mean, RMSE: root mean square error). Bold entries represent statistical significance.

NRS, Numerical Rating Scale; PASS, Pain Anxiety Symptoms Scale.

### 3.8. Group differences of other measures related to pain

As shown in Table 5, the mixed-effects model revealed time and group effects and their interaction effect, which represents the effect of surgery relative to conservative care from visit 1 to 3. There was no statistically significant surgical effect on the MPQ, ODI, PCS, PASS, PANAS, or BDI. The Conservative group showed greater improvement of the PSQ compared to the PELD (PSQ/min: *t*_4,266_ = 2.07, *P* = 0.039; PSQ/mod: *t*_4,266_ = 2.38, *P* = 0.018).

**Table 5:** Group differences of other measures related to pain.

	Time	Group	Time × Group
	*t*-value, *P*	*t*-value, *P*	*t*-value, *P*
NRS	−4.16, <0.001	0.78, 0.439	−0.86, 0.391
MPQ/vas	−5.52, <0.001	0.09, 0.929	−0.14, 0.891
MPQ/a	−0.51, 0.612	1.74, 0.083	−1.77, 0.078
MPQ/s	0.06, 0.952	0.34, 0.735	−1.17, 0.242
ODI	−3.00, 0.003	1.79, 0.074	−0.65, 0.517
PCS/r	−2.70, 0.007	0.13, 0.900	−0.54, 0.589
PCS/m	−2.46, 0.015	−1.08, 0.280	0.43, 0.670
PCS/h	−1.64, 0.102	0.28, 0.779	−0.82, 0.415
PASS/f	−2.24, 0.026	−0.84, 0.401	0.35, 0.726
PASS/p	0.33, 0.742	−0.24, 0.809	−0.59, 0.555
PASS/c	−2.23, 0.027	−1.09, 0.279	0.47, 0.638
PASS/e	−2.14, 0.034	0.49, 0.626	−0.49, 0.625
PANAS/n	−0.12, 0.908	1.18, 0.238	−0.81, 0.420
PANAS/p	−0.94, 0.350	−0.05, 0.961	−0.80, 0.423
BDI	−1.89, <0.001	−0.51, 0.614	−0.10, 0.918
PSQ/min	−3.03, 0.003	−2.88, 0.004	2.07, **0.039**
PSQ/mod	−4.30, <0.001	−2.71, 0.007	2.38, **0.018**

Interaction effect of time and group revealed that there was no statistically significant surgical effect on the MPQ, ODI, PCS, PASS, PANAS, or BDI. The Conservative group showed greater improvement of the PSQ compared with the PELD (PSQ/min: *t*_4,266_ = 2.07, *P* = 0.039; PSQ/mod: *t*_4,266_ = 2.38, *P* = 0.018). Bold entries represent statistical significance.

BDI, Beck Depression Inventory; NRS, Numerical Rating Scale; MPQ, McGill Pain Questionnaire; ODI, Oswestry Disability Index; PCS, Pain Catastrophizing Scale; PASS, Pain Anxiety Symptoms Scale; PANAS, Positive and Negative Affect Schedule; PSQ, Pain Sensitivity Questionnaire; PELD, percutaneous endoscopic lumbar discectomy.

## 4. Discussion

This study, by virtue of using an exponential pain reduction model applied to daily momentary pain ratings for 3 months, revealed statistically significant benefits of PELD compared with conservative treatments in terms of both rapidity and 3-month pain recovery. Rapid pain relief is a consistent outcome of the surgery as shown in a number of studies; however, outcomes after 3 months do not statistically significantly differ from conservative treatments.^16,39^ Percutaneous endoscopic lumbar discectomy is a less invasive surgery with the advantages of causing less damage, using local anesthesia, and is associated with fast patient recovery compared with other discectomy procedures,^40^ suggesting the adverse effects of lumbar discectomy can be minimized. Indeed, PELD seems to result in rapid pain relief of leg and/or low back pain after the surgery, which is maintained for 3 months.^29^

Pain evaluation in this study was different from other studies, which typically sample just a few time points during the recovery phase. However, pain after the surgery fluctuates due to a number of factors, including nocebo or placebo effects, daily change of mood, recurrence of herniation, socioeconomic change, switching of primary doctors and/or hospitals, follow-up treatments, and even attitude of the staff who conducted the pain survey. As a result, these fluctuations would greatly increase variability—and decrease the reliability—of longitudinal pain measurements. To answer the question of “does long-term pain relief reflect the surgical effect?,” the continuous daily momentary pain rating via a smartphone app was able to quantify fluctuations across the whole pain recovery period. Furthermore, with the assistance of the exponential pain reduction model, the unique features of pain reduction could be derived from each subject's time series, allowing for greater accuracy when evaluating the surgical effect compared with conservative pain management.

An interesting phenomenon was observed in our study (Table 2), in that NRS reported on site was statistically significantly greater than both averaged 3-day pain rating and estimated pain from the model, while the latter 2 pain measures were not statistically different. The discrepancy between NRS and daily pain ratings^5^ may contribute to patients' overestimation of pain intensity when they visited their doctor and completed pain questionnaires. The overestimation bias may be a reason why several previous studies did not demonstrate long-term effectiveness of the discectomy. In this regard, daily pain ratings or pain estimated from a model may be a better candidate to measure pain intensity.

The application of exponential pain reduction models to fit pain recovery trajectories assumes that the pain time course could be characterized by time-dependent exponential decay with unique parameters for each patient during the first 90 days after treatments (PELD or Conservative).^19^ Although some clinicians may consider this assumption clinically unacceptable, it seems to allow for the elimination of noise in pain assessments to better quantify treatment effectiveness, and also to account for missing data.^19^ As long as the condition during the first few months does not change dramatically, the model may keep predicting the actual pain in the future. Therefore, fitted functions should be a reliable way to evaluate the surgical effectiveness.

### 4.1. Limitations

First, although this study has revealed benefits of endoscopic discectomy by assessing daily momentary pain with a 2-parameter pain reduction model, we cannot exclude the possibility that there may be addition confounding by indication that was not addressed by the IP of treatment weighting. Such factors may include, for example, selection bias based on variables that were not included, IP model misspecification, no control for neuropathic pain conditions,^17^ lack of allocation concealment and lack of blinding of participants that may induce a placebo effect, and nonstandardized conservative treatment. That such confounding factors may be present limits the clinical significance of these findings, but they do lend evidence and a rationale for a rigorous, randomized experiment.

Second, we analyzed back pain and leg pain together, because LDH is considered to be an etiology of both sites of pain, and clinically, many symptomatic patients with LDH have pain in both sites. However, the distribution of the dominant pain site was different between PELD and Conservative groups. Approximately 80% of participants who underwent PELD had dominant leg pain as compared to only 47% in Conservative group. Unfortunately, the sample size of Conservative group was too small to separate participants based on pain sites.

Third, 11-point NRS might not be an adequate measure for individuals who showed pain recovery. Because a floor effect of the NRS would be greater in individuals with lower pain intensity before the surgery, the rapid pain reduction could be identified easily in those with higher pain. However, this finding is consistent with many previous studies,^16,39^ suggesting the floor effect is considered to be little.

Fourth, in this study, there was no daily functional assessment like daily activity although disability is an indicator of the surgical effectiveness and treatment success, and the daily pain assessment did not discern spontaneous pain not involving physical activity and pain related to movement, but the latter may be a particularly important measure of musculoskeletal pain in PELD patients when they gradually recovered from surgery.^10^ In future studies, by using the smartphone app developed in this study, which provides a possibility to collect the day-to-day variability of activities accompanied with movement-evoked pain, the movement-evoked pain could be distinguished from the daily pain ratings. Such a possibility would enable the exploration of the relationship between activity and both types of pain.

## 5. Conclusions

In conclusion, both momentary pain rating and the exponential pain reduction model fitting revealed that PELD provided rapid pain recovery that was maintained for at least 3 months compared with conservative treatments. Greater pain intensity and lower anxiety before the surgery were predictors of faster pain reduction in the recovery subgroup after PELD. Daily momentary pain rating on a smartphone may be able to provide more informative data to evaluate effect of an intervention than pain assessment on hospital visits.

## Disclosures

The authors have no conflicts of interest to declare.
